# Can a translational science approach change the dementia narrative in medical education?

**DOI:** 10.3389/frdem.2023.1288817

**Published:** 2023-11-24

**Authors:** Alison Warren

**Affiliations:** The Department of Clinical Research and Leadership, George Washington University School of Medicine and Health Sciences, Washington, DC, United States

**Keywords:** Alzheimer's disease, personhood, pathology, dementia, medical education, stigma

## Introduction

The field of translational science, while still nascent, has emerged to train future translational researchers to efficiently narrow the gap between science-to-service (Corcoran et al., 2019). A more efficient “bench to bedside” approach is necessary for a multitude of conditions and public health policies, including dementia (Wehling, 2015). The National Center for Advancing Translational Sciences (NCATS) offers a model that places the patient at the center of research, implementation, and dissemination domains (U.S. Department of Health and Human Services National Institutes of Health, 2021). Incorporating this kind of model into basic medical education from a person-centered, integrated, trans-disciplinary perspective may add considerable value not only to advanced biomedical treatment options (Woolf, 2008), but also to the person-centered care (PCC) needed (Mohr et al., 2021) by over 55 million people around the world (2023 Alzheimer's disease facts figures, 2023).

## Dementia-related stigma in healthcare

Stigma of any kind ultimately renders the sufferer as one who is tainted, discounted, and devalued (Goffman, 1974). The considerable contribution of stigma to illness burden and social exclusion indicates its importance as a clinical issue (Lyons and Dolezal, 2017). Older adults are often marginalized by perceptions of incompetence and diminished global abilities (Kane, 2002; Stites et al., 2018). These disparities are evident in cognitively intact individuals, as with the case of ageism, and even more so in persons who experience cognitive decline (Kane, 2002; Stites et al., 2018). Notably, some literature has suggested that perceived stigma toward Alzheimer's disease is higher among healthcare professionals than the public (Piver et al., 2013). In medicine, stigma can delay timely diagnosis and compromise successful diagnosis, treatment, and health outcomes (Nyblade et al., 2019). For example, a provider's reluctance to communicate a dementia diagnosis or lack of referral to a specialist, and decreased quality of life; depression; and suicide in the patient (Bacsu et al., 2020). Despite the recent increased awareness of dementia-related stigma, stigma-reduction interventions remain paltry (Bacsu et al., 2022). Furthermore, medical professionals may not have a thorough understanding of dementia pathology. The Alzheimer's Disease International (ADI) conducted the largest global survey in 2019 on attitudes to dementia that included 70,000 people across 54 countries and found that 62% of healthcare practitioners believe dementia is a normal part of aging, suggesting a need to improve the understanding of dementia as a medical condition in healthcare professionals as well as the general public (Lynch, 2020). Additionally, Chan et al. (2022) conducted a cross-sectional study involving 464 final year medical students from seven universities in Malaysia and found that overall dementia knowledge of respondents with and without exposure was low. Increased awareness and education during the early stages of medical education may help to bridge this gap (Tullo and Gordon, 2013; Chan et al., 2022). Considering the complexity of dementia care (Dreier-Wolfgramm et al., 2017), addressing stigma via “a translational approach that integrates across multiple levels of analysis, from basic science to intervention research” (Querdasi and Callaghan, 2023, p. 104) may facilitate a person-centered culture to reduce dementia-related stigma in medicine.

## Personhood and pathology in the classroom

There is a great deal of variability in dementia education. Certain specialties, such as geriatrics, neuropsychiatry, and neuropsychology impart advanced education about the care needs of the aging population, but this education is not uniform across specialties. The literature suggests that medical students excel in diagnostic assessment of dementia, while nursing students exhibit more positive attitudes and preference for PCC compared to medical and pharmacy students (Basri et al., 2017; Scott et al., 2019). Medical students are taught important basics of dementia epidemiology and pathophysiology, but humanistic considerations are lacking (Griffiths et al., 2020), such as addressing needs through nonverbal communication in persons with dementia who have compromised verbal abilities (Wood et al., 2017). Impaired verbal communication in persons with dementia, along with difficulty interpreting nonverbal communications by carers, is a common mutual source of frustration (Wood et al., 2017). Another example is pain management, which is not included in required topics covered in medical school curricula, yet an online learning module developed by the University of Pittsburgh Center of Excellence in Pain Education administered to third-year medical students demonstrated significant benefit (Moehl et al., 2020). Online modules that address the unique needs of persons with dementia can be implemented with relative ease.

A study by Tullo and Gordon (2013) sought to evaluate the quality of medical education about dementia using a survey including 23 medical schools across the United Kingdom. All provided some dementia-specific education regarding general knowledge but were lacking in education about behaviors and attitudes (Tullo and Gordon, 2013). Further analysis showed that only 80% of schools incorporated formal assessment of dementia-specific learning outcomes and there was a widespread deficiency in engagement with multidisciplinary teams, patients, and carers in teaching (Tullo and Gordon, 2013). To address negative attitudes, a novel study of an Enhanced Geropsychiatric Experience (EGE) in a dementia facility for third year medical students was conducted by Blazek et al. (2016). Students were given the opportunity to examine patients in their living environment without the time constraints of a more typical clinical environment. The EGE consisted of two full days of students working with an interprofessional team to examine, assess, and recommend treatment plans during the first full day, and were able to observe the results of their recommendations 3 weeks later during the second full day. Post EGE, the students reported their experiences positively affected their comfort level and attitudes toward persons with dementia (Blazek et al., 2016). A recent experiential study was conducted by Goldman and Trommer (2019) in which 101 premedical students participated *in A Friend for Rachel* program which was designed to reduce dementia-related stigma and foster understanding by providing sustained exposure to persons with dementia. This study required students to write weekly reflections about their interactions with persons with dementia, the analysis of which demonstrated an increased appreciation for the complexities of living with dementia and the need for compassionate care in this patient population (Goldman and Trommer, 2019). Based on the need to better prepare healthcare professionals to effectively and respectfully communicate with persons with dementia, Wood et al. (2017) designed an educational program that combined class-based sessions with experiential learning in dementia care homes. Students were provided the opportunity to practice communication skills with persons with dementia (Wood et al., 2017). Questionnaires used to assess student experience showed positive responses reflecting that their confidence, communication, and sense of preparedness improved (Wood et al., 2017).

PCC emphasizes the treatment of patients as whole persons rather than the sum of their symptoms (Kitwood, 1997). The concept of placing the person in the driving position of their healthcare decisions has become the gold standard in recent years, and the use of the more wholistic term “person-centered” rather than “patient-centered” has been suggested by an expert panel of the American Geriatrics Society (Brummel-Smith et al., 2016). Built on the work of Carl Rogers, Tom Kitwood applied the concept of PCC to persons with dementia, which has served as a major influence in nursing education (Mitchell and Agnelli, 2015), and has been embraced in many national guidelines and dementia plans (Downs and Lord, 2017; Mohr et al., 2021). Many programs in memory care centers have been highly influenced by PCC that have led to the creation and implementation of dementia care models but are not yet ubiquitous (Røsvik et al., 2011). However, research to improve the application of PCC in nursing continues to evolve to meet the critical need for specialized dementia care professionals, where innovative educational strategies are still lacking (Kulla and Slettebø, 2023). A recent scoping review of 154 studies examining the experience of persons with dementia and their formal caregivers' use of Kitwood's framework in institutional settings demonstrated benefit in the vast majority of studies (Terkelsen et al., 2020). Some geriatric medical fellowships are also beginning to incorporate PCC into their curriculum, such as the University of California San Francisco (University of California San Francisco Geriatrics, 2023), and others are offering specialized graduate programs in aging and health (e.g., Georgetown University's Master of Science in Aging and Health; Arizona State University Master of Science in Aging) to promote PCC and facilitate the dissolution of the social injustice incurred by older adults (Georgetown University Master of Science in Aging Health, 2022).

## Addressing biases in healthcare education

The onerous vicissitudes of research and high demands of clinical care can, at times, inadvertently create a myopic medicalized lens focused on pathology, rather than the person (Heap and Wolverson, 2020). University curricula is an opportunity to address age-related biases and misconceptions to positively impact health equality (Scott et al., 2019), and elucidate preserved capacities, such as emotions (Lee et al., 2019) and implicit memory (Sabat, 2006). Medicalized discourses overshadow interpersonal communication that may leave one feeling disregarded, disempowered, dehumanized, and degraded (Heap and Wolverson, 2020). Not as a function of malcontent, but through unintentionally damaging behaviors toward persons with dementia, such as infantilization, stigmatization, outpacing, invalidation, and banishment (Kitwood, 1993).

Negative attitudes and stigma appear to stem from lack of education (Blazek et al., 2016; Scott et al., 2019; Griffiths et al., 2020; Chan et al., 2022), lack of clinical encounters (Blazek et al., 2016; Goldman and Trommer, 2019; Griffiths et al., 2020; Chan et al., 2022) and/or vicarious adoption of negative attitudes from clinical supervisors (Ayu et al., 2022). The attitudes of medical faculty are an important aspect of education and role modeling, and can either contribute to stigma or facilitate dignity. Studies utilizing didactic and clinical programs to enhance education and direct contact demonstrate an improvement in attitudes, increased empathy, and reduction in stigma (Blazek et al., 2016; Goldman and Trommer, 2019; Gonella et al., 2019; Griffiths et al., 2020). Short educational sessions of just 1 h have shown improvement of positive attitudes and perceptions toward persons with dementia (Griffiths et al., 2020). Williams and Daley (2021) conducted a scoping review to examine the healthcare education models being used to promote PCC regardless of specialty. Programs used for comparison included long-term experiential; activity-centered with people with dementia; interprofessional education; immersive conference style; and dementia simulation models. They found that the majority of programs demonstrated improvement in student knowledge, confidence, and positive attitudes, with the highest efficacy seen in programs involving direct contact with people living with dementia (Williams and Daley, 2021).

The language used by medical professionals to speak with and about patients can be inadvertently offensive and stigmatizing, specifically with the widespread use of the term “patient.” Utilizing respectful, dementia-friendly language can avoid the association of behaviors with an inherent problem with the person (i.e., using terms such as “person with dementia,” “person living with dementia”; “person diagnosed with dementia,” and avoiding terms such as “demented,” “victim,” “burden”) (Heap and Wolverson, 2020). Dementia friendly initiatives have been emerging to move beyond the lens of pathology to foster well-being, human rights, and social inclusion for persons with dementia (Hebert and Scales, 2019).

## Conclusion

The knowledge and attitudes about dementia of students in healthcare are especially important, as they are the next generation who carry the responsibility to care for the growing aging population (Basri et al., 2017). Unfortunately, many medical providers tend to overfocus on an imminent decline and demise following a dementia diagnosis, swiftly prescribing end of life plans (Swaffer, 2014). Using a translational science approach to education that incorporates dementia-friendly initiatives and PCC can broaden the medicalized lens from which dementia is often viewed (Hebert and Scales, 2019) to promote quality of life. Indeed, “it is necessary to understand more than the anatomical, neurophysiological, and neurochemical losses that characterize AD” (Sabat, 2021, p. 233). Literature supports that the inclusion of didactic and practical education improves quality of care, clinical outcomes, and cost savings (Reuben et al., 2023). Prioritizing an integrative translational approach to medical education could dramatically reduce mental, emotional, and physical suffering. In short, it is imperative to redefine medical education to emphasize the retained abilities in dementia, address the stigma surrounding dementia, and improve communication strategies for professionals and caregivers.

## Author contributions

AW: Writing—original draft.
